# Incidence and survival of HNSCC patients living with HIV compared with HIV-negative HNSCC patients

**DOI:** 10.1007/s00405-020-06573-9

**Published:** 2021-01-25

**Authors:** Katharina Haase, Iris Piwonski, Carmen Stromberger, Nadine Thieme, Max Heiland, Benedicta Beck-Broichsitter, Veit M. Hofmann, Grzegorz Kofla, Steffen Sander, Ullrich Keilholz, Konrad Neumann, Katharina Stölzel, Heidi Olze, Philipp Arens, Steffen Dommerich, Annekatrin Coordes

**Affiliations:** 1 Department of Otorhinolaryngology, Head and Neck Surgery, Charité—Universitätsmedizin Berlin, corporate member of Freie Universität Berlin, Humboldt-Universität Zu Berlin, and Berlin Institute of Health, Campus Virchow Klinikum and Campus Charité Mitte, Augustenburger Platz 1, 13353 Berlin, Germany; 2 Department of Pathology, Charité—Universitätsmedizin Berlin, corporate member of Freie Universität Berlin, Humboldt-Universität Zu Berlin, and Berlin Institute of Health, Berlin, Germany; 3 Department of Radiooncology, Charité—Universitätsmedizin Berlin, corporate member of Freie Universität Berlin, Humboldt-Universität Zu Berlin, and Berlin Institute of Health, Campus Virchow Klinikum, Berlin, Germany; 4 Department of Radiology, Charité—Universitätsmedizin Berlin, corporate member of Freie Universität Berlin, Humboldt-Universität Zu Berlin, and Berlin Institute of Health, Campus Virchow Klinikum, Berlin, Germany; 5 Department of Oral and Maxillofacial Surgery, Charité—Universitätsmedizin Berlin, corporate member of Freie Universität Berlin, Humboldt-Universität Zu Berlin, and Berlin Institute of Health, Campus Virchow Klinikum and Campus Benjamin Franklin, Berlin, Germany; 6 Department of Otorhinolaryngology, Head and Neck Surgery, Charité—Universitätsmedizin Berlin, corporate member of Freie Universität Berlin, Humboldt-Universität Zu Berlin, and Berlin Institute of Health, Campus Benjamin Franklin, Berlin, Germany; 7 Department of Oncology, Charité—Universitätsmedizin Berlin, corporate member of Freie Universität Berlin, Humboldt-Universität Zu Berlin, and Berlin Institute of Health, Campus Virchow Klinikum, Berlin, Germany; 8 Clinical Cancer Registry, Charité Comprehensive Center (CCCC), Charité—Universitätsmedizin Berlin, corporate member of Freie Universität Berlin, Humboldt-Universität Zu Berlin, and Berlin Institute of Health, Campus Charité Mitte, Berlin, Germany; 9 Department of Oncology, Charité—Universitätsmedizin Berlin, corporate member of Freie Universität Berlin, Humboldt-Universität Zu Berlin, and Berlin Institute of Health, Campus Benjamin Franklin, Berlin, Germany; 10 Institute for Biometrics and Clinical Epidemiology, Charité—Universitätsmedizin Berlin, corporate member of Freie Universität Berlin, Humboldt-Universität Zu Berlin, and Berlin Institute of Health, Campus Charité Mitte, Berlin, Germany

**Keywords:** HNSCC, HIV, Survival, Smoking, p16

## Abstract

**Purpose:**

The aim was to analyze the incidence and survival of patients living with HIV (PLWH) with head and neck squamous cell carcinoma (HNSCC) and to compare with a control group of HIV-negative HNSCC patients.

**Methods:**

Clinicopathological data and predictors for overall survival (OS) and disease-free survival (DFS) were investigated (2009–2019).

**Results:**

50 of 5151 HNSCC patients (0.97%) were PLWH, and 76% were smokers. Age ≤ 60 years, HIV-PCR ≤ 50 copies, CD4 cells ≤ 200/mm^3^, cART treatment, T and UICC classification, oral cavity and nasal/paranasal sinuses, and therapy were significantly associated with OS in univariate analysis. In the multivariate analysis, only age and HIV-PCR independently predicted OS. The OS of the 50 PLWH was not significantly altered compared with the 5101 HIV-negative controls. However, OS and DFS were significantly inferior in advanced tumor stages of PLWH compared with an age-matched control group of 150 HIV-negative patients.

**Conclusions:**

PLWH were diagnosed with HNSCC at a significantly younger age compared to HIV-negative patients. Taking into account patient age at initial diagnosis, both OS and DFS rates in PLWH are significantly worse compared with a matched control group of HIV-negative patients in advanced tumor stages UICC III/IV. The prognosis (OS) is improved when taking cART treatment, the HIV viral load is undetectable and CD4 count is high.

**Supplementary Information:**

The online version contains supplementary material available at 10.1007/s00405-020-06573-9.

## Introduction

The prevalence of HIV infections in Germany is 0.1% [1]. HIV infections impair lymphocyte function and are therefore implicated in decreased tumor surveillance and increased cancer pathogenesis. Since the introduction of antiretroviral therapy in 1996, AIDS defining malignancies (ADMs) have declined, especially Kaposi sarcoma and non-Hodgkin lymphoma [2, 3]. HIV is now a chronic infection and people live with HIV (PLWH). However, the decrease of ADMs has been associated with an increase in non-AIDS defining malignancies (NADMs), which include lung, anal, liver, and head and neck carcinomas [4]. Compared to the age-matched general population, NADM are more common in PLWH [5, 6]. Oncogenic viruses contribute to cancerogenesis, e.g., Human Papillomaviruses (HPV) and Epstein–Barr viruses (EBV).

Head and neck squamous cell carcinoma (HNSCC) is the sixth most common cancer worldwide with an annual global incidence of 500,000 [7, 8]. Major risk factors are excessive alcohol and tobacco consumption [9]. The proportion of smokers is higher in PLWH compared with the general population [10, 11]. Therefore, PLWH and smokers may create an overlapping group. Another major risk factor for the development of HNSCC generally is persistent infections with high-risk HPV, especially HPV16 [12]. HPV infections are two-to-six times more common in PLWH [13]. HIV promotes the penetration of HPV viruses into the epithelium [14]. In PLWH, 40% of all malignancies are attributable to viral infections (compared with 4% in the general population) [15]. Thus, HPV may be an important risk factor for PLWH without a history of tobacco or alcohol consumption.

HIV is traditionally an exclusion criterion for clinical cancer trials. Therefore, a few publications are available on treatment and outcomes of PLWH and HNSCC. Picard et al*.* published the data of 47 Paris patients who were initially diagnosed with HNSCC between 1994 and 2014 [16]. Mourad et al*.* provide data of 73 New York patients with an initial HNSCC diagnosis between 1997 and 2010 [17]. Further investigations from the United States have included approximately 40 PLWH and HNSCC [18] which were compared with HIV-negative HNSCC [19–21]. Two studies including 15 and 24 PLWH and HNSCC (1995–2011 and 1985–1994) compared the survival outcomes with a control group of 3503 and 515 HIV-negative HNSCC patients [22, 23]. The French HIV study group investigated 248 patients treated in 17 centers with HNSCC (2004–2014) [24] and the North American AIDS Cohort Collaboration investigated 286 patients treated in 17 centers (1996–2009) [25].

The objective of the current study was to investigate the incidence of PLWH with HNSCC in Berlin (Germany) and to compare the long-term survival with both a control group of 5101 HIV-negative HNSCC patients and an age-matched control group of 150 patients, respectively. In addition, we investigated the impact of several clinicopathological factors on overall survival (OS) and disease-free survival (DFS) in PLWH taking into account the patient's age.

## Materials and methods

### Patient inclusion criteria

Following Institutional Review Board approval (appl.no.EA420117), data were reviewed from all patients with histologically confirmed head and neck malignancies (larynx, oro/naso/hypopharynx, oral cavity, and nasal/paranasal sinuses) who underwent diagnostic procedures and/or therapy at the current center between 2009 and 2019. Clinicopathological data of PLWH who developed solid HNSCC were evaluated in detail.

### Patient and treatment assessment

The assessment of the 50 PLWH diagnosed with HNSCC included medical history, physical examination, serum laboratory tests, and imaging studies. A suitable control group included 150 HIV-negative HNSCC patients. Three control patients were assigned to one PLWH, taking into account the patient age (± 1–2 years), UICC stage, and gender. According to the tumor stage and tumor site of every patient, the individual therapeutic approaches were discussed by a multidisciplinary tumor board (head and neck surgeons, medical oncologists, radiation oncologist, and head and neck radiologists), considering internationally recognized and established treatment standards. Surgical treatment required clinical in-sano resectability assessment. Neck dissection was always performed during the same procedure. All resected specimens were examined histologically. R0 resection included complete tumor removal with microscopically negative surgical margins without tumor cells. Adjuvant RT (radiotherapy) or CRT (chemoradiation) was performed in patients with advanced tumor stage (UICC > II), histological evident disease (R1) or close surgical margins status, and extra capsular lymph-node spread. In locally advanced tumor stages (UICC III and IV), a definitive RT/CRT was considered as an alternative by the multidisciplinary tumor board, or the only treatment option if the tumor was deemed unresectable. The tumor stage was documented using AJCC 8th edition depending on the initial diagnosis. To investigate the impact of HPV, the surrogate marker p16 was used.

### Immunohistochemistry

In all formalin fixed and paraffin-embedded tissue samples, the squamous cell carcinoma content was estimated by hematoxylin and eosin staining of the tissue sections (2 µm). All samples with < 10% tumor content were excluded from p16 analysis. Immunohistochemical staining was performed using BenchMark ULTRA autostainer (Ventana, Tucson, Arizona, USA), the monoclonal rabbit antibody p16INKA4 (CINtec Histology Kit; Ventana Medical Systems, Inc.1910E. Innovation Park Drive Tucson, Arizona 85,755) according to the manufacturer’s instructions. Overexpression of p16 was defined as medium to strong (2+/3+) intensity of the nuclear staining with a distribution of ≥ 75% of the tumor cells.

### HIV diagnostics

HIV diagnostics included PCR testing of viral load and quantitative estimation of CD4 and CD8 cells. CD4 counts and CD4/CD8 ratio were used as a marker for the current integrity of the patient´s immune status.

### Statistical analysis

Continuous variables with normal distribution were presented as mean with standard deviation according to SAMPL Guidelines [26]. Nominal variables were expressed as number and percentage. To compare patient age, we used T test after controlling for normal distribution.

The primary outcomes were the incidence of HNSCC in PLWH and the OS and DFS after initial diagnosis of the HNSCC in PLWH compared with HIV-negative patients. OS and DFS were defined as time between the initial diagnosis of the HNSCC and the date of death or last follow-up and the date of tumor recurrence, respectively, using the Kaplan–Meier method. For univariate analyses, log-rank tests were used to assess significance.

The following clinicopathological variables were recorded and analyzed: sex (male *versus* female), age (≤ 60 *versus* > 60 years), time between initial diagnosis of HIV and initial diagnosis of HNSCC (< 18 *versus* ≥ 18 years), history of smoking (positive *versus* negative), alcohol abuse (yes *versus* no), HIV viral load (≤ 50 *versus* > 50 copies), CD4 cells (≤ 200 *versus* > 200 copies), cART treatment (yes *versus* no), p16 detection (positive *versus* negative), additional cancers (yes *versus* no), T classification (T1 *versus* T2 *versus* T3 *versus* T4), N classification (positive *versus* negative), UICC classification (I/II *versus* III/IV), tumor grading (G1 *versus* G2 *versus* G3), tumor site (larynx *versus* oral cavity *versus* oropharynx *versus* hypopharynx versus nasal/paranasal sinuses *versus* nasopharynx), and tumor therapy (surgery only *versus* surgery + adjuvant RT/CRT *versus* RT/CRT *versus* palliative care).

All survival-associated variables (*P* < 0.05) in the univariate analysis were further investigated using the Cox multivariate regression model with backward elimination. *P* values < 0.05 were considered statistically significant. Significant collinearity factors were excluded by calculating the variance inflation factor (VIF) using linear regression analysis when values were below 5. Significant differences in Cox regression were checked for normal distribution of the residuals by Schoenfeld´s test. Possible influences of single unknown or missing data were assessed by additional performance of the multiple imputation model (five cycles, Mersenne twister random number generator). This revealed no relevant changes in significance parameters compared to the non-imputed data. Statistical analyses were performed using the SPSS software package, version 25.2 (SPSS, IBM Corp., Armonk, NY, USA).

## Results

### Patient characteristics

During the study period, 50 of 5151 HNSCC patients were PLWH (0.97%). The clinicopathological data are summarized in Table 1. The mean age at the initial diagnosis of HIV was 37 years (23–59 years), and the mean time between initial diagnosis of HIV and initial diagnosis of HNSCC was 18 years (0–35 years). The mean age at the initial diagnosis of HNSCC in PLWH was 55 years (35–71), which was significantly less compared to the HIV-negative HNSCC patients (62 years (29–95), *p* < 0001). 92% of the HNSCC PLWH were male, 76% were smokers, and 40% were drinkers.

**Table 1:** Characteristics of patients with head and neck squamous cell carcinoma living with HIV infection (*n* = 50)

Variable	*n* = 50
Mean age at initial diagnosis of HNSCC, years (SD, range)	54.64 (8.275, 35–71)
Mean age at initial diagnosis of HIV, years (SD, range)	37.00 (9.495, 23–59)
Mean time between initial diagnosis of HIV and initial diagnosis of HNSCC, years (SD, range)	17.90 (8.497, 0–35)
Male (%)	46 (92)
Smoking (%)	38 (76)
Alcohol abuse (%)	20 (40)
Additional cancers (%)	
Overall	12 (24)
AIDS-related	4
Non-AIDS-related	8
HPV-related	1
HNSCC characteristics	
Tumor site	
Oropharynx (%)	19 (38)
Oral cavity (%)	17 (34)
Larynx (%)	4 (8)
Hypopharynx (%)	4 (8)
Nasal/paranasal sinuses (%)	4 (8)
Nasopharynx (%)	2 (4)
P16 HNSCC (%)	
Positive	17/38 (45)
P16 Oropharynx-Ca (%)	
Positive	12/16 (75)
Grading	
G1 (%)	2 (4)
G2 (%)	41 (82)
G3 (%)	7 (14)
T classification (T)	
T1 (%)	17 (34)
T2 (%)	10 (20)
T3 (%)	9 (18)
T4 (%)	14 (28)
N classification (%)	
Positive	21 (42)
M classification (%)	
Positive	0 (0)
UICC	
I (%)	12 (24)
II (%)	5 (10)
III (%)	8 (16)
IV (%)	25 (50)
Therapy	
Surgery only (%)	16 (32)
Pall. Surgery only (%)	1 (2)
Surgery + RT/CRT (%)	10 (20)
CRT (%)	20 (40)
Palliative/best supportive care (%)	3 (6)
HIV characteristics	
Viral load, HIV-PCR (%)	
Not detectable	32 (64)
Median CD4 cell count (cells/μl)	300 (10–1255)
Median CD8 cell count (cells/μl)	715 (70–1760)
CD4/CD8 ratio	
< 0.5 (%)	24 (48)
CDC Stage 1	0 (0)
CDC Stage 2	30 (60)
CDC Stage 3	20 (40)
Under cART (%)	
(%)	40 (80)
Under PI (%)	
(%)	14 (28)
Coinfection	
HBV (%)	10 (20)
HCV (%)	6 (12)
HBV and HCV (%)	5 (10)

*SD* standard deviation; *ART* antiretroviral therapy; *PI* protease inhibitor; *HBV* hepatitis B virus; *HCV* hepatitis C virus; *CDC* HIV classification system of the United States Centers for Disease Control

### Head and neck squamous cell carcinoma

**Fig. 1:**
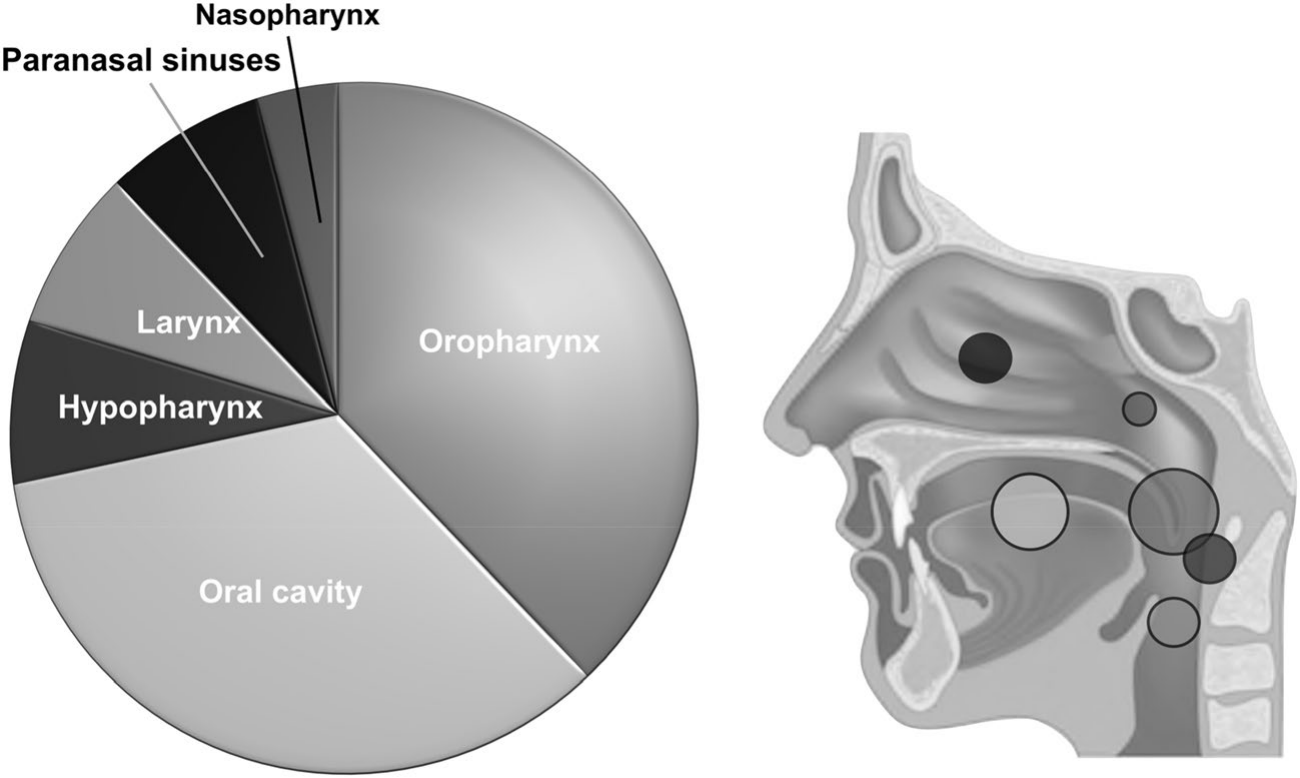
Tumor sites of the 50 patients with head and neck squamous cell carcinoma and HIV infection. The pie chart presents the proportional percentage of the tumor subsites

The head and neck malignancies were located in the oropharynx (*n* = 19, 38%), oral cavity (*n* = 17, 34%), larynx (*n* = 4, 8%), hypopharynx (*n* = 4, 8%), nasopharynx (*n* = 2, 4%), and nasal/paranasal sinuses (*n* = 4, 8%) (Fig. 1). All tumors in the HIV group were histologically confirmed squamous cell carcinoma (50/50). The majority were moderately differentiated (*n* = 41, 82%). At the time of the initial cancer diagnosis, 34% of patients were at an early tumor stage (UICC I and II) and 66% were advanced (UICC III and IV). Based on tumor stage, tumor resection only was performed on 17 patients. Ten patients underwent surgery followed by adjuvant treatment, definitive CRT/RT occurred in 20 patients and palliative/best supportive care in three patients. In 7 of the 24 surgically treated patients, histological evaluation revealed microscopically positive surgical margins. RT was performed with 64-72 Gy, and systemic therapy regimens included five fluorouracil and cisplatin plus/minus 5-Fluorouracil. Impaired medical conditions prevented two patients from receiving concurrent chemotherapy RT.

The control group of HIV-negative patients with HNSCC included 5101 patients spread over comparable tumor subsites and located in the oropharynx (31%), oral cavity (36%), larynx (18%), hypopharynx (8%), nasopharynx (3%), and nasal/paranasal sinuses (5%).

### HIV characteristics

In 32 of 50 PLWH (64%), viral load at initial diagnosis of HNSCC was not detectable. A CD4 count ≤ 200/mm^3^ was observed in 18 of 50 PLWH (36%). A CD4/CD8 ratio < 0.5% was found in 24 PLWH (48%). 40 of 45 PLWH received cART treatment (89%; six were unknown), which included in 14 cases HIV protease inhibitor (PI) treatment (31%). Co-infections of hepatitis B virus (HBV) were found in 10 of 47 tested patients (21%), hepatitis C virus (HCV) in 6 of 47 tested patients (13%), and simultaneous HBV/HCV in five of 47 tested patients (11%). P16 was investigated in 38 of the 50 PLWH with HNSCC. 17 of the 38 patients were p16 positive (45%). In PLWH and oropharynx carcinoma, 16 of 19 were investigated and 12 of the 16 patients were p16 positive (75%).

### Long-term survival of PLWH

**Fig. 2:**
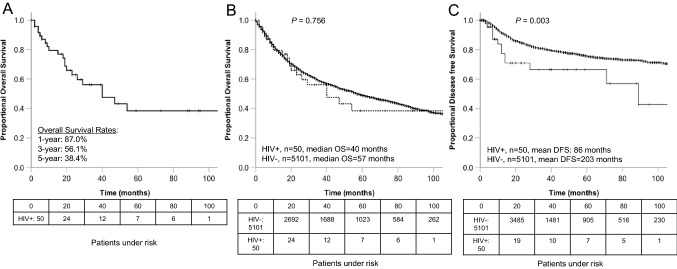
Survival: **a** overall survival (OS) of the 50 PLWH with HNSCC. **b** OS of the 50 PLWH and HNSCC compared with 5101 HIV-negative patients with HNSCC. **c** Disease-free survival (DFS) of the 50 PLWH with HNSCC compared with 5101 HIV-negative patients with HNSCC. The boxes below the graphics show the patients at risk

After a median follow-up time of 20 (0–211) months after initial cancer diagnosis in PLWH, the median survival was 40 months (95%CI 16.0–63.9). The 1, 3, and 5 year OS rates of these patients were 87.0, 56.1, and 38.4% respectively (Fig. 2A). During the follow-up period, 21 of the 50 patients died. In ten patients, the death was cancer related, and nine of those were at an advanced tumor stage (UICC III and IV) at initial diagnosis.

### Predictors of survival

The results of univariate and multivariate analyses as predictors of OS and DFS for PLWH are summarized in Table 2. In univariate analysis, prognostic factors positively associated with OS included being younger than 60 at initial diagnosis of HNSCC (*p* = 0.005), ≤ 50 copies in HIV-PCR (*p* = 0.005), CD4 cell count > 200 (*p* < 0.001), under cART treatment (*p* = 0.005), early tumor stage in T classification (*p* = 0.006) and UICC classification (*p* = 0.012), tumor sites oral cavity (*p* = 0.046), and nasal/paranasal sinuses (*p* = 0.002) and tumor therapy (surgical treatment only vs. all other treatments, *p* = 0.023). In the multivariate analysis, only an age of ≤ 60 years at diagnosis and HIV-PCR < 50 copies at initial HIV diagnosis (HR = 11.58, CI 95% 2.06–64.92, *P* = 0.005) independently predicted OS.

**Table 2:** Univariate and multivariate analyses of clinicopathologic variables associated with overall survival (OS) and disease-free survival (DFS) in 50 patients with head and neck carcinoma and HIV infection

		Overall survival (OS)			Disease free survival (DFS)	
		Univariate analysis		Multivariate analysis	Univariate analysis	
Variable	*N* = 50	Mean OS (months)	*P*	*P*, HR (CI 95%)	Mean DFS (months)	*P*
Age (years)						
≤ 60	39	99	0.001 V:1.15	0.026, 0.276 (0.089–0.860) ^1)^	90	0.236
> 60	11	22			44	
Time between initial diagnosis of HIV and initial diagnosis of HNSCC (years)						
< 18	19	55	0.273		86	0.535
≥ 18	21	55			69	
Sex						
Male	46	84	0.621		89	0.556
Female	4	32			31	
Smoking						
Positive	38	80	0.863		89	0.200
Negative	9	26			15	
Alcohol abuse						
Yes	26	69	0.967		120	0.044
No	20	48			49	
HIV-PCR						
≤ 50 copies	32	97	0.005 V:1.33	0.007, 6.954 (1.698–28.488) ^2)^	107	0.274
> 50 copies	18	33			56	
CD4 cells						
≤ 200	18	28	< 0.001 V:1.54	0.546	61	0.615
> 200	32	107			94	
cART treatment						
Yes	40	77	0.005 V:1.44	0.906	97	0.460
No	4	11			12	
PI treatment						
Yes	14	56	0.546		42	0.123
No	29	64			112	
P16 HNSCC (%)						
Positive	17	44	0.645		65	0.665
Negative	21	107			71	
Additional cancers						
Yes	12	32	0.128		91	0.263
No	38	90			44	
T classification						
T1	17	120	0.006	0.058	84	0.852
T2	10	54	0.690		67	0.539
T3	9	18	0.067		17	0.572
T4	14	18	0.004	0.790	31	0.987
N classification						
≥ 1	21	50	0.823		61	0.660
0	29	80			92	
UICC						
I/II	17	113	0.012 V:4.11	0.643	96	0.570
III/IV	33	37			60	
Grading						
G1	2	19	0.509		8	0.138
G2	41	80	0.855		84	0.876
G3	7	55	0.891		80	0.635
Tumor site						
Oropharynx	19	47	0.708		65	0.847
Oral cavity	17	107	0.046	0.650	86	0.900
Larynx	4	22	0.521		30	0.473
Hypopharynx	4	24	0.806		6	0.340
Nasal/paranasal sinuses	4	9	0.002	0.599	13	0.357
Nasopharynx	2	8	0.072		2	0.705
Therapy						
Only surgery	16	31	0.023 V: 3.59	0.206	92	0.885
Other treatment	34	17			63	

Significant p values are underlined

*ART* antiretroviral therapy; *PI* protease inhibitor; *HNSCC* head and neck squamous cell carcinoma; *V* Variance Inflation Factor

Schoenfeld´s test 1) *p* = 0.894; 2) *p* = 0.045

P16 did not have any significant impact on OS in PLWH (17/38 (45%) were p16 + ; *p* = 0.645), even in the small subgroup of PLWH with oropharynx carcinoma (16/19 (84%); *p* = 0.436). However, in HIV-negative patients with oropharynx carcinoma, a significant effect of the p16 status on OS was observed (p16 positive: n = 269, meanOS: 56 months; p16 negative: *n* = 139, meanOS: 50 months;*P* = 0.036).

### Long-term survival of PLWH compared with controls

The survival of HIV-negative HNSCC patients (*n* = 5101) was not significantly different (median OS 57 months, 95% CI 52.1–61.9, *p* = 0.756, Fig. 2B, Table 3). A comparable result was found for the four most common tumor subsites: oropharynx, oral cavity, larynx, and hypopharynx. Again, there was no significant difference of the OS between PLWH and HIV-negative patients (Supplementary Fig. 1). However, the DFS of PLWH was significantly diminished compared to the non-HIV population [27] (*p* = 0.003, Fig. 2c), especially in UICC III/IV (*p* = 0.001).

**Table 3:** Univariate analysis of clinicopathologic variables associated with overall survival (OS) and disease-free survival (DFS) in 50 patients with head and neck malignancy and HIV infection compared to 5101 HIV-negative patients

Variable			Overall survival			Disease free survival		
	HIV+ (*N* = 50)	HIV− (*N* = 5101)	Mean OS HIV + (months)	Mean OS HIV-(months)	*P*	Mean DFS HIV + (months)	Mean DFS HIV-(months)	*P*
All patients	50	5101	80	98	0.756	86	203	0.003
Female	4	1413	32	86	0.318	31	131	0.111
Male	46	3688	84	101	0.939	89	218	0.009
Smokers	38	1106	80	82	0.681	89	106	0.213
Non-smokers	9	591	26	66	0.522	15	73	0.072
Localization								
Oropharynx	19	1589	47	87	0.480	65	183	0.014
Oral cavity	17	1840	84	107	0.155	86	136	0.087
Larynx	4	899	22	127	0.231	30	216	0.205
Hypopharynx	4	407	23	42	0.494	n.e	n.e	0.541
Paranasal sinus	4	233	81	9	0.001	13	108	0.163
Nasopharynx	2	133	88	8	0.051	n.e	n.e	0.920
P16 +	17	374	44	58	0.215	65	65	0.518
P16-	21	458	107	57	0.849	71	64	0.498
Grade 1	2	293	19	143	0.120	8	207	0.001
Grade 2	41	2690	80	87	0.988	84	165	0.013
Grade 3	7	1177	55	73	0.870	80	134	0.618
UICC I/II	17	827	113	124	0.617	147	174	0.504
UICC III/IV	33	2410	37	65	0.652	60	158	0.001

Significant p values are underlined

**Fig. 3:**
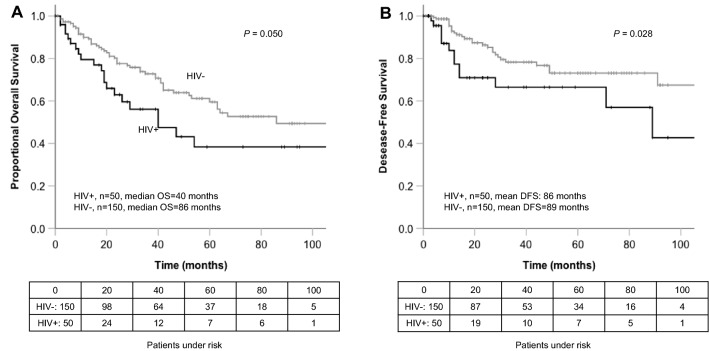
Survival compared to an age-matched control group. Overall survival (**a**) and disease-free survival (**b**) of the 50 PLWH and HNSCC compared with a matched control group of 150 HNSCC patients taking in account patient age, UICC stage, tumor site, and subordinate gender (Supplementary Fig. 2, Table 4)

As we found a significant difference in patient age at initial HNSCC diagnosis in PLWH compared with HIV-negative patients, we performed an additional matched-pair analysis (1:3 match) taking into account patient age, UICC stage, and tumor site (Table 4, Supplementary Fig. 2) which significantly impacted OS in PLWH. Gender matching was considered subordinate when possible. However, it was not possible in small subgroups of less frequent sub-localisations (nasopharynx, hypopharynx, and paranasal sinuses) and in patients with tumor stage UICC I. In the matched-pair analysis, both OS (OS for all patients: *p* = 0.050; for UICC III/IV stages *p* = 0.010, Fig. 3a, Table 4) and DFS (DFS for all patients: *p* = 0.028; for UICC III/IV stages *p* = 0.010 Fig. 3b, Table 4) were significantly reduced in PLWH.

**Table 4:** Univariate analysis of clinicopathologic variables associated with overall survival (OS) and disease-free survival (DFS) in 50 patients with head and neck squamous cell carcinoma (HNSCC) and HIV infection compared to 150 matched HIV-negative patients taking into account patient age at initial diagnosis of HNSCC, UICC stage, tumor site, and subordinate gender. Complete Gender matching was not possible in small subgroups (nasopharynx, hypopharynx, paranasal sinuses, UICC I)

Variable			Overall survival					Disease-free survival				
	HIV + (*n* = 50)	HIV- (*n* = 150)	Mean OS HIV + (months)	SD	Mean OS HIV-(months)	SD	*P*	Mean DFS HIV + (months)	SD	Mean DFS HIV-(months)	SD	*P*
All patients	50	150	80.3	13.4	96.4	6.8	0.050	86.2	13.5	89.2	4.3	0.028
Female sex	4	24	32.0	6.5	84.9	10.9	0.150	31.0	9.0	73.7	12.7	0.654
Male sex	46	126	84.5	14.4	94.9	7.2	0.135	89	14.2	91.6	4.5	0.026
Smokers	38	42	79.8	14.3	62.3	6.6	0.114	88.8	14.1	79.9	5.4	0.060
Non-smokers	9	19	26.0	4.0	74.6	12.3	0.249	14.7	4.4	76.9	11.4	0.067
Localisation												
Oropharynx	19	57	46.8	9.9	62.1	6.3	0.348	65.3	11.1	90.2	6.0	0.109
Oral cavity	17	51	107.4	21.2	135.0	9.9	0.213	85.9	19.8	86.9	7.2	0.250
Larynx	4	12	21.5	8.8	58.2	11.1	0.545	30.0	7.8	67.8	11.0	0.414
Hypopharynx	4	12	24.0	7.3	46.9	7.4	0.692	n.e		n.e		n.e
Paranasal sinus	4	12	8.5	2.8	29.9	7.1	0.214	12.7	3.5	26.6	5.0	0.464
Nasopharynx	2	6	8.0	0.0	90.8	14.9	0.014	n.e		n.e		n.e
P16 +	17	10	43.6	8.4	73.3	12.0	0.101	64.8	11.3	31.3	5.3	0.433
P16−	21	15	107.3	19.3	40.6	21.1	0.882	n.e		n.e		n.e
Grade 1	2	4	19	7.8	21	10.2	0.157	8	0.7	21	10.2	0.317
Grade 2	41	81	80.1	14.3	100.7	8.9	0.094	83.5	14.9	88.9	5.9	0.076
Grade 3	7	30	54.9	21.3	76.2	8.6	0.422	79.8	13.8	79.5	9.1	0.856
UICC I/II	17	51	113	20.5	98.4	6.4	0.175	96.4	19.8	90.7	7.2	0.421
UICC III/IV	33	99	37.5	8.1	82.4	8.0	0.010	60.3	9.4	84.4	5.0	0.010

## Discussion

This study analyzed the incidence and survival of PLWH who developed HNSCC. After reviewing the data of 5151 patients with HNSCC who were treated in our center between 2009 and 2019, we identified 50 PLWH (1%) who were diagnosed with HNSCC, 100% histologically confirmed as HNSCC.

The OS of PLWH did not significantly differ from HIV-negative patients. However, patient age at initial HNSCC diagnosis was significantly younger compared to HIV-negative HNSCC patients. Therefore, after PLWH and HNSCC were matched with a control group based on patient age, tumor site, UICC stage, and subordinated gender, a significant difference was found in both OS and DFS, each in the advanced UICC stages III/IV.

Very few studies in the literature have investigated PLWH and HNSCC. The largest cohort includes 286 and 248 patients treated in multiple centers in France and North America, respectively, without an HIV-negative control group comparison [24, 25]. Two smaller studies including 15 and 24 PLWH and HNSCC compared survival with an HIV-negative control group [22, 23]. The current study is the largest monocenter study of PLWH 10 years after cART therapy. It compared survival rates with HIV-negative patients according to sub-localization and tumor stage, and it compared PLWH and HNSCC with a matched control group taking in account patient age, UICC stage, tumor site, and gender.

The characteristics of the patients included in this study were comparable with those of PLWH in previous studies reporting on HNSCC. 92% were males, mean time between initial HIV diagnosis and initial HNSCC diagnosis was 17.9 years [16], and the mean age at initial HNSCC diagnosis was significantly earlier compared to HIV-negative patients [4, 16]. The most frequent tumor sites were oropharynx (38.0%) and oral cavity (34.0%) [16]. Two older studies found the larynx to be the most frequent subsite [22, 28]. In the current study, 76% were smokers and 49% had a history of alcohol abuse. PLWH have an increased risk profile developing HNSCC. PLWH smoke more often compared with the general population and have greater difficulty quitting smoking [10, 11]. Therefore, PLWH with high-risk TP53 mutations have poor survival outcomes and the fastest development of distant metastases [20, 21]. Another major risk factor for the HNSCC pathogenesis are persistent infections with high-risk HPV, especially HPV16 [12, 23]. In PLWH and HNSCC, the heterogeneity of HPV subtypes is higher [16, 20]. In the general population, persistent HPV infections may cause 25% of HNSCC and up to 75% of oropharyngeal cancer [12]. Long-term immunosuppression increases the risk of malignancies associated with oncogenic viral infections. Ceccarelli et al. systematically investigated the correlation of HPV and HIV in HNSCC patients [29]. In the current study, p16 did not have a significant impact on OS in PLWH (*p* = 0.645) and in the small subgroup of PLWH with oropharynx carcinoma (*p* = 0.436). However, 16 out of 19 patients (84%) had p16 positive oropharynx carcinoma and it is reasonable to assume that HPV contributes to the development of oropharynx carcinoma in PLWH. The p16 negative group was probably too small to show statistical significance. In HIV-negative patients with oropharynx carcinoma, p16 was associated with significantly improved OS (*p* = 0.036) which is consistent with the literature [30–32]. Therefore, the 8th edition of UICC presented a different tumor staging depending on the detection of p16. This classification does not consider smoking habits. In PLWH with p16 positive oropharynx carcinoma who are smokers, survival classified by the risk of death score proposed by Ang et al. [33] (which is based on HPV status and smoking history) may provide a better correlation. In the current study, 10 of 29 (35%) PLWH had p16 positive HNSCC and were smokers, and seven of ten (70%) had oropharyngeal carcinoma. Additionally, current smokers have significantly higher HPV infections [34].

In PLWH, HNSCC are more frequent compared to the general population. Beachler et al*.* [25] found a threefold increased incidence of HNSCC in PLWH and Robbins et al. found a doubled incidence in oral cavity and pharyngeal carcinoma in PLWH in the United States [35]. In the current study, we found a tenfold increased prevalence of HIV in HNSCC patients compared to the general population [1]*.*

In the current study, the treatment strategies depended on the UICC stage (Supplementary Fig. 1). 85% of patients in the current study with advanced tumors (UICC III and IV) were treated with resection combined with adjuvant RT/CRT or primary RT/CRT only, whereas 88% of patients at an early tumor stage (UICC I and II) underwent resection only. Patients eligible for surgery only had a significantly improved OS compared to patients whose tumor stage required surgery combined with adjuvant RT/CRT or primary RT/CRT (*P* = 0.023**)**. The latter two therapeutic options did not reveal a significant difference in OS (*P* = 0.278).

Picard et al., who investigated PLWH between 1997 and 2010 in New York, concluded that PLWH have worse OS and DFS undergoing definitive R(C)T compared with HIV-negative HNSCC patients [17]. The poorer outcomes in both OS and DFS in PLWH with advanced tumor stages which require adjuvant or primary RT/CRT could be explained by the fact that long-term immunosuppression by the virus may impair the therapeutic treatment’s action [36, 37].

In addition to the increased risk profile of many HIV patients for HNSCC, recent work from the United States has shown that PLWH have a distinct HNSCC mutation pattern [38]. Oncogenic HIV may also promote its own pathogenesis of HNSCC which is attributed to the HIV transactivator protein *tat* which stimulates the cell cycle and inhibits apoptosis [39]. Additionally, *tat* upregulates the expression of the oncoproteins E6 and E7 [40]. This may explain the results of cohort studies which showed that HIV infection may increase lung cancer risk after adjusting for tobacco and other confounders [41, 42].

In the current study, patients with > 50 copies of HIV-PCR (*p* = 0.005) and a CD4 cell count ≤ 200 cells/mm^3^ had a significantly poorer prognosis (*p* < 0.001). A low number of CD4 + T lymphocytes may support the carcinogenesis of HNSCC [25] and may be associated with poor prognosis [19]. Other groups showed HPV-positive status was associated with a CD4 + nadir of < 200, but not with CD4 + level at time of diagnosis [16].

Immune checkpoint inhibitors are promising cancer therapies. In PLWH, very little experience is available at present. An ongoing trial is evaluating pembrolizumab in PLWH and a variety of metastatic cancers. Inclusion criteria are having a CD4 + T-cell count of > 200 (NCT02595866). However, HIV is no contraindication for treatment with anti PD-1. Patients with HIV/AIDS and low CD4 + T-cell counts should be monitored closely [43]. Liu et al. have shown that HIV PI sensitize HNSCC cells to radiotherapy by activation of endoplasmic reticulum stress and induction of an immunogenic cell death. Therefore, HIV PIs may be potentially used in combination with radiation in the treatment of HNSCC [44]. In the current study, cART treatment had a significant impact on OS, while the application of HIV PIs in 31% did not alter OS. However, only 7/14 patients with cART and HIV PIs underwent definitive or adjuvant (C)RT. Therefore, further investigations are necessary. By triggering the immunogenic cell death, the PI therapy could also increase the effect of the immune checkpoint inhibitors.

Our current study of PLWH who developed HNSCC has some limitations. Given the rarity of this disease in PLWH (50 of 5151 patients, 0.97%), compiling a cohort large enough to facilitate prognostic factor analysis (see Table 4) would require including patients from an even longer time period or comparing multiple centers including a control group of HIV-negative HNSCC patients.

## Conclusion

PLWH were diagnosed with HNSCC at a significantly younger age compared to HIV-negative patients. Taking into account patient age at initial diagnosis, both OS and DFS rates in PLWH are significantly worse compared with a matched control group of HIV-negative patients in advanced tumor stages UICC III/IV. The prognosis (OS) is improved when taking cART treatment, the HIV viral load is undetectable and CD4 count is high.

## Supplementary Information

Below is the link to the electronic supplementary material.

Supplementary file1 (PPTX 3282 KB)

Supplementary file2 (PPTX 8443 KB)

## Data Availability

All relevant data are transparent presented, supplemental figures include additional information of the patient cohort and the matched group.

## Code availability

SPSS software package, version 25.2 (SPSS, IBM Corp., Armonk, NY, USA).
