# Supplementary material for: Incidence and survival of HNSCC patients living with HIV compared with HIV-negative HNSCC patients
Source: Eur Arch Otorhinolaryngol. 2021 Jan 25;278(10):3941–53. doi: 10.1007/s00405-020-06573-9 (PMC8382606; doi:10.1007/s00405-020-06573-9)
Supplement: Supplementary file 1 — Supplementary file1 (PPTX 3282 KB) [file 405_2020_6573_MOESM1_ESM.pptx]

## Slide 1
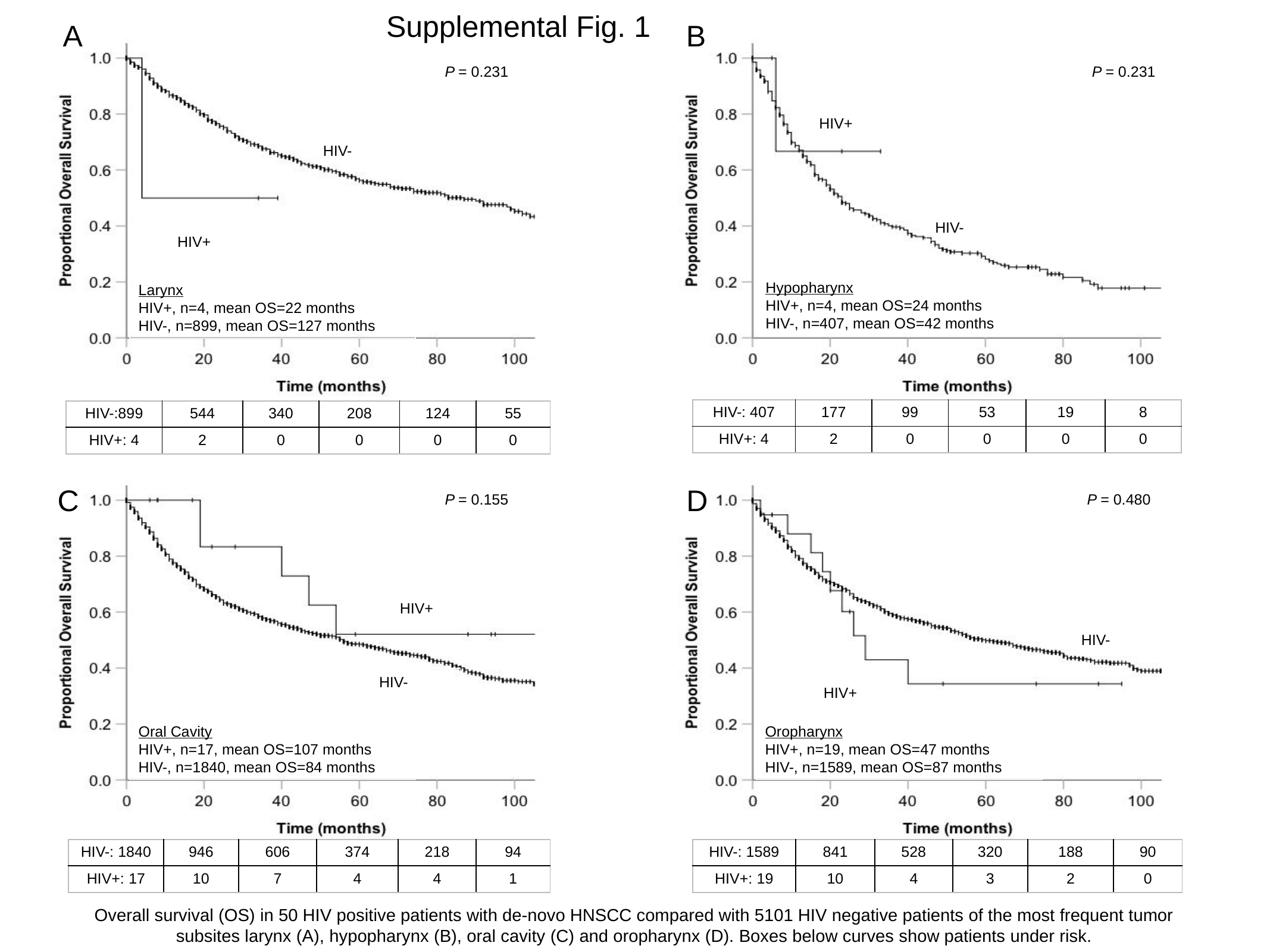

Supplemental Fig. 1
A
B
P = 0.231
P = 0.231
HIV+
HIV-
HIV-
HIV+
Hypopharynx
HIV+, n=4, mean OS=24 months
HIV-, n=407, mean OS=42 months
Larynx
HIV+, n=4, mean OS=22 months
HIV-, n=899, mean OS=127 months
| HIV-: 407 | 177 | 99 | 53 | 19 | 8 |
| --- | --- | --- | --- | --- | --- |
| HIV+: 4 | 2 | 0 | 0 | 0 | 0 |
| HIV-:899 | 544 | 340 | 208 | 124 | 55 |
| --- | --- | --- | --- | --- | --- |
| HIV+: 4 | 2 | 0 | 0 | 0 | 0 |
C
D
P = 0.155
P = 0.480
HIV+
HIV-
HIV-
HIV+
Oropharynx
HIV+, n=19, mean OS=47 months
HIV-, n=1589, mean OS=87 months
Oral Cavity
HIV+, n=17, mean OS=107 months
HIV-, n=1840, mean OS=84 months
| HIV-: 1840 | 946 | 606 | 374 | 218 | 94 |
| --- | --- | --- | --- | --- | --- |
| HIV+: 17 | 10 | 7 | 4 | 4 | 1 |
| HIV-: 1589 | 841 | 528 | 320 | 188 | 90 |
| --- | --- | --- | --- | --- | --- |
| HIV+: 19 | 10 | 4 | 3 | 2 | 0 |
Overall survival (OS) in 50 HIV positive patients with de-novo HNSCC compared with 5101 HIV negative patients of the most frequent tumor subsites larynx (A), hypopharynx (B), oral cavity (C) and oropharynx (D). Boxes below curves show patients under risk.
