# Supplementary material for: Incidence and survival of HNSCC patients living with HIV compared with HIV-negative HNSCC patients
Source: Eur Arch Otorhinolaryngol. 2021 Jan 25;278(10):3941–53. doi: 10.1007/s00405-020-06573-9 (PMC8382606; doi:10.1007/s00405-020-06573-9)
Supplement: Supplementary file 2 — Supplementary file2 (PPTX 8443 KB) [file 405_2020_6573_MOESM2_ESM.pptx]

## Slide 1
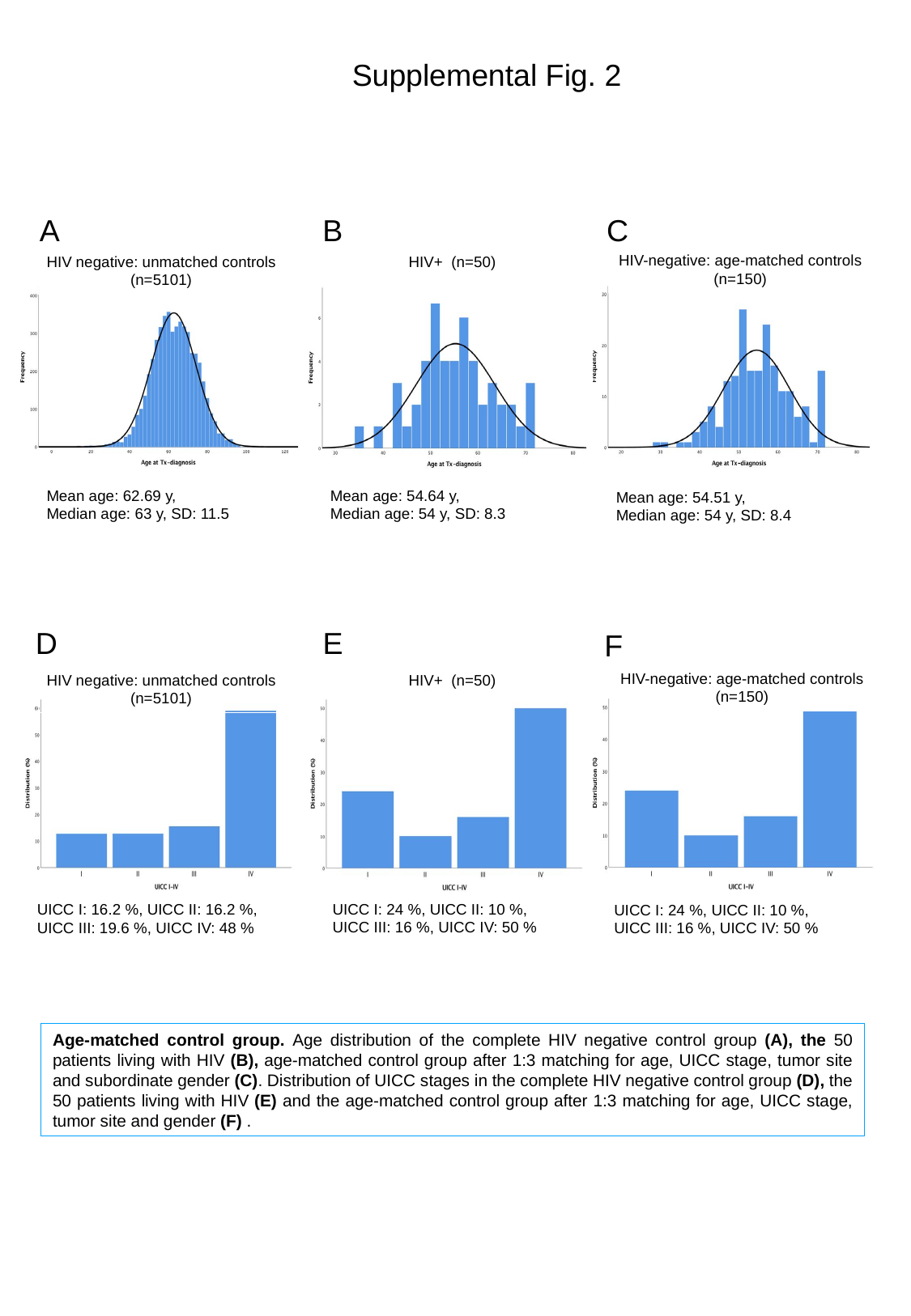

Supplemental Fig. 2
B
C
A
HIV-negative: age-matched controls (n=150)
HIV negative: unmatched controls (n=5101)
HIV+ (n=50)
Mean age: 62.69 y,
Median age: 63 y, SD: 11.5
Mean age: 54.64 y,
Median age: 54 y, SD: 8.3
Mean age: 54.51 y,
Median age: 54 y, SD: 8.4
D
E
F
HIV-negative: age-matched controls (n=150)
HIV negative: unmatched controls (n=5101)
HIV+ (n=50)
UICC I: 24 %, UICC II: 10 %,
UICC III: 16 %, UICC IV: 50 %
UICC I: 16.2 %, UICC II: 16.2 %,
UICC III: 19.6 %, UICC IV: 48 %
UICC I: 24 %, UICC II: 10 %,
UICC III: 16 %, UICC IV: 50 %
Age-matched control group. Age distribution of the complete HIV negative control group (A), the 50 patients living with HIV (B), age-matched control group after 1:3 matching for age, UICC stage, tumor site and subordinate gender (C). Distribution of UICC stages in the complete HIV negative control group (D), the 50 patients living with HIV (E) and the age-matched control group after 1:3 matching for age, UICC stage, tumor site and gender (F) .
